# Case report of an unusual presentation of *Staphylococcus aureus* induced toxic shock syndrome/hyperimmunoglobulinemia E syndrome

**DOI:** 10.1097/MD.0000000000019746

**Published:** 2020-04-10

**Authors:** Harry S. Jacob, Gregory M. Vercellotti, Donald Y.M. Leung, Patrick M. Schlievert

**Affiliations:** a Department of Medicine, University of Minnesota, Minneapolis, MN; b Department of Pediatrics, National Jewish Health, Denver, Colorado and Department of Pediatrics, University of Colorado at Denver Health Sciences Center, Aurora, CO; c Department of Microbiology and Immunology University of Iowa, Iowa City, IA.

**Keywords:** hyperimmunoglobulin E syndrome, *Staphylococcus aureus*, toxic shock syndrome

## Abstract

**Rationale:**

Toxic shock syndrome (TSS) typically is an acute onset multi-organ infection caused by TSS toxin-1 producing *Staphylococcus aureus.* Herein we describe a highly unusual case report.

**Patient concerns:**

A male patient self-referred to the University of Minnesota Hospital with a chronic history of *S aureus* infection with accompanying fever, hypotension, and nonhealing, football-sized lesion on his leg.

**Diagnosis:**

An unusual case presentation of TSS/hyperimmunoglobulin E syndrome is described. The patient had a leg wound from which TSS toxin-1 *S aureus* was isolated. The patient exhibited characteristic skewing of T cells to those with variable region, β-chain T cell receptor-2. Other patients have been seen with related presentations.

**Interventions:**

The following therapeutic regimen was instituted: vigorous antibacterial scrubs several times daily plus intravenous Ancef 3 days each month; intravenous infusions of immunoglobulin G infusions (28 gm) every 3 weeks; and weekly subcutaneous injections of recombinant granulocyte colony-stimulating factor.

**Outcome:**

Improvement was obvious within 3 months: no further cellulitic episodes occurred; the patient regained 95 pounds in 9 months; blanching and cyanosis of fingers disappeared within 3 months as did intractable pain although mild hypesthesias continued for 2 years; erythroderma resolved, and repeat skin biopsies performed after 2 years no longer demonstrated T cell receptor skewing. Although IgE levels have not completely returned to normal, the patient remains in excellent health.

**Lessons:**

We propose that staphylococcal TSST-1 was responsible for the serious problems suffered by this patient as suggested by the following features: rapid onset of chronic, life-threatening, disorder that began with an episode of staphylococcal sepsis; the extraordinary elevation of IgE levels in this previously non-atopic individual; the acquired severe granulocyte chemotactic defect that accompanied this hyperimmunoglobulinemia (“Job Syndrome”) with its accompanying wound-healing defect; and the striking diffuse erythroderma, including palmar erythema (“Red Man Syndrome”) with hypotension and fever that also characterizes TSS.

## 1 Introduction

Although syndromes such as scarlet fever and toxic shock syndrome (TSS; staphylococcal- or streptococcal-induced) are well-accepted examples of superantigen (SAg)-mediated disorders,^[1]^ other, often sporadic and previously-mysterious illnesses, also likely involve SAg toxicity.^[1]^ The following case, recapitulated almost exactly by 3 others seen recently at the University of Minnesota Medical School, exemplifies this presumption.

## 2 Case report

Institutional Review Board approval for this case report is not needed. However, the authors have patient approval for its presentation. G.S., a 54-year-old businessman, entered hospital with high fever (104.6°F), prostration (blood pressure 80/40), severe finger pain, and obvious cellulitis of the left forearm. During the past 4 years, he had been admitted to multiple institutions over 30 times with similar symptoms and signs. Coagulase-positive *Staphylococcus aureus* had been isolated from blood and cellulitic lesions on numerous occasions. Cellulitis without abscess formation was a constant feature, and, in fact, an attempt to “drain” an inflamed thigh lesion 2 years previously produced catastrophic necrosis of most of the posterior thigh soft tissue, eventuating in a football-sized nonhealing wound open down to the muscle layer. This defect was refractory to all wound-healing therapeutic efforts and had manifested no epithelialization over the preceding 2 years.

The patient appeared cachectic (he had lost 95 pounds over the past 3 years) and manifested remarkable erythroderma diffusely over the face, palms, and soles with more patchy macular red areas over the trunk and shoulders. The fingers of both hands were exquisitely painful with cyanotic as well as dead-white patches. One terminal phalanx was frankly gangrenous and ultimately self-amputated. The skin over the dorsum of hands and wrists was thickened and mimicked that of scleroderma; periorbital skin was wrinkled, atrophic, and reminiscent of that of chronic atopic disease. Alopecia areata, especially of the temporal areas, was prominent.

Relevant laboratory data included: blood smears that demonstrated mild granulocytopenia (absolute neutrophil count approximately 1500/mm^3^) with toxic granulation and Dohle Bodies; sporadic Sezary-type lymphoid cells were also noted; immunoglobulins were normal except for an extraordinary elevation of IgE (2500–3000 mg/dL vs normal 700–1600 mg/dL); serologies for known connective tissue diseases were negative; and complement levels were not reduced. Blood cultures were negative, but swabs from axillae, groin, perianal regions, and throat grew virtually pure cultures of *S aureus*, which were subsequently shown to produce abundant amounts of toxic shock syndrome toxin-1 (TSST-1). After cellulitis had cleared following intravenous anti-staphylococcal therapy, a skin chemotaxis assay (Rebuck Skin Window technique) demonstrated severely-deficient neutrophil chemotaxis (3–5 PMN/high power field versus greater than 100/high power field in windows from healthy volunteers).

At this juncture a more rigorous medical history was obtained. The patient had been perfectly well, weighing 205 pounds (height was 6′ 1′′) with no previous hospitalizations or serious illnesses until 4 years before this admission. He dates his rapid downhill course to an attempt by a physician to percutaneously drain an apparent bursitis of the elbow. High fever, rapidly spreading infection with bacteremia followed, and required a 2-week in-patient hospitalization for intravenous antibiotic administration. Thereafter, the patient developed innumerable episodes of cellulitis involving multiple areas (including the aforementioned massive thigh necrosis). These episodes required hospitalizations at roughly-monthly intervals and were associated with striking weight loss; the patient became house-bound and unable to work. Six months before admission to the University of Minnesota Hospital, he developed progressive Raynaud symptomatology, progressing to continuous excruciating pain that required Fentanyl patches and resisted all therapy including bilateral wrist Clonidine patches. In addition to the wound-healing deficit, the patient developed spontaneous and life-threatening ulceration of the proximal esophagus with perforation and associated acute mediastinitis. Of possible further significance (vide infra), it was learned that the patient's only daughter suffers severe, episodic psoriasis which worsens with respiratory streptococcal infections, termed “guttate psoriasis.”^[2]^

The patient was diagnosed with acquired Hyperimmunoglobulin E Syndrome (“Job Syndrome”), but of a presumed, previously-undescribed etiology – that of chronic staphylococcal superantigenemia. To buttress this presumption, skin biopsies of the macular, plaque-like lesions were analyzed. Although a marked proliferation of dermal lymphocytes suggested mycosis fungoides, pathognomomic Pautrier microabcesses were not observed. Nevertheless, immunohistologic assay detected a marked predominance of T helper cells that do characterize this disorder. Molecular subtyping of T cell receptors performed by author DYML, validated striking variable region, β-chain T cell receptor skewing (greater than 70% Vβ2 T cells) in several biopsy specimens. Such skewing of Vβ2 T cells from a normal value of approximately 10% of T cells to the observed 70% is characteristic of TSST-1 SAg effects.^[3]^

With seemingly-reasonable support for the proposition that this patient's unique syndrome might be driven by the staphylococcal pyrogenic toxin SAg TSST-1, the following therapeutic regimen was instituted: vigorous antibacterial scrubs (with Physohex) several times daily plus intravenous Ancef 3 days each month. Moreover, the recognition (vide infra) that Kawasaki Disease may be TSST-1 mediated^4^,^5^ and can be rapidly ameliorated/cured with intravenous infusions of immunoglobulin G, stimulated us to provide similar infusions (28 gm) every 3 weeks. Finally, in an attempt to amplify numbers and chemotactic efficiency of granulocytes, we provided weekly subcutaneous injections of recombinant granulocyte colony-stimulating factor.

Improvement was obvious within 3 months; no further cellulitic episodes occurred; the patient began gaining weight (regaining 95 pounds to his usual habitus in 9 months); blanching and cyanosis of fingers disappeared within 3 months as did intractable pain – although mild hypesthesias continued for 2 years; erythroderma resolved, and repeat skin biopsies performed after 2 years no longer demonstrated lymphocyte accumulation or Vβ2 T cell receptor skewing. Although IgE levels has not completely returned to normal, the patient remains in excellent health and is fully-employed 2.5 years’ later on no therapy other than Viagra.

## 3 Discussion

That staphylococcal SAg(s) were responsible for the serious problems suffered by this patient is suggested by the following features:

(1) the rapid onset of his chronic, yet life-threatening, disorder that began with an episode of severe staphylococcal sepsis;
(2) the extraordinary elevation of IgE levels in this previously non-atopic individual;
(3) the acquired severe granulocyte chemotactic defect that accompanied this hyperimmunoglobulinemia (“Job Syndrome”) with its accompanying wound-healing defect; and
(4) the striking diffuse erythroderma, including palmar erythema (“Red Man Syndrome”) with hypotension and fever that also characterizes TSS.^6^,^7^,^8^,^9^

Favoring our postulate is:

(1) bacteriologic documentation of generalized skin colonization with TSST-1-producing *S aureus*;
(2) the accumulation of T helper cells in affected dermis with marked skewing of T cell receptor Vβ2 chains expected with TSST-1 SAg stimulation; and
(3) his complete recovery following intensive, prolonged anti-staphylococcal therapy plus IgG infusions.

We suggest that this patient may not be unique. With perceptions heightened by his syndrome, we have recently identified 3 other patients with similar clinical and bacteriologic findings on our medical services; all have recovered with identical therapeutic maneuvers. We suggest this case may provide insights into other clinical disorders of previously-uncertain etiology. Examples include:

(1) Mycosis fungoides and scleroderma.^[10]^ In the former instance the cutaneous lymphoproliferative disorder specifically involves T helper cells and characteristically remains indolent for several years but abruptly becomes a lethal disseminated lymphoma. In fact, the remarkable generalized erythroderma along with erythematous plaque-like lesions in our patient suggested this diagnosis initially. We speculate that mycosis fungoides, at least in some instances, might reflect chronic stimulation of dermal T helper cells by skin-colonizing, SAg-producing staphylococci (or perhaps streptococci); the resulting prolonged oligoclonal proliferation, we reason, would favor new, more oncogenic mutations. In fact, a similar hypothesis has recently been supported by provocative findings of Vβ T cell receptor skewing in mycosis fungoides patients, where patients were found to harbor TSST-1-producing *S aureus*, and astonishingly, a percentage of these went into prolonged remission with anti-staphylococcal therapy.^[10]^
(2) We were consulted on 2 cases of sudden patient death with isolation of TSST-1 positive *S aureus* from mucosal surfaces in both cases. These patients had exceptionally-high cardiac eosinophilia upon autopsy by Dr Lee Wattenberg (now deceased) at the University of Minnesota upon autopsy. He suggested these patients succumbed to anaphylaxis enhanced by TSST-1 induced Vβ2 skewing of T cells to T helper 2 type T cells with elevated IgE to one or more staphylococcal antigens.

Confirmatory studies are awaited.

The other diagnosis initially entertained in our patient was scleroderma. Severe Raynaud symptoms coupled with typical skin thickening over dorsal surfaces of hands and forearms supported this diagnosis, although serologic tests were not confirmatory. Intriguingly, recent studies strongly buttress the proposition that scleroderma is a chronic T cell aggressing disease. That is, chronic graft-versus-host disease, that may follow bone marrow transplantation, mimics idiopathic scleroderma closely, and recent provocative findings have demonstrated that women with this disorder often harbor long-lived, activated memory T cells derived from their (male) children; this makes rational a postulate that scleroderma is often due to (fetal) graft-versus-host disease. We suggest that, in some cases, it might also be driven by chronic superantigenemia. If so, it seems likely that its microvascular compromise might involve in some way cytokine release from activated T cells. For instance, TNF (cachexin; note this patient's cachexia), released by TSST-1 exposed T cells, is vasoconstricting.^[1]^ In addition, recent studies of skin vessels from laboratory animals chronically injected intradermally with staphylococcal or streptococcal SAgs demonstrate intraluminal aggregation and vessel wall infiltration by lymphocytes^[1]^; moreover, we have personally noted in peripheral blood smears of mycosis fungoides patients that Sezary cells tend to be aggregated. Thus, both cytokine-mediated microvascular spasm plus vaso-occlusion attending aggregation of activated lymphocytes, may have caused the digit loss in our patient, as well as be involved in the Raynaud phenomena of idiopathic scleroderma.

Recent data also support the role of SAgs in other syndromes. As noted Kawasaki Syndrome, a lymphocytic macrovasculitis, may involve TSST-1 and other SAgs; that is, sera from KD patients are unable to inhibit TSST-1 activation of T lymphocytes in vitro; addition of pooled IgG corrects the defect in vitro and cures the disease when infused intravenously; and many Kawasaki Syndrome patients are chronically colonized with TSST-1 producing *S aureus.* ^2^,^5^,^11^,^12^,^13^,^14^ Finally, we are intrigued that it has been proposed that “guttate psoriasis” may reflect (streptococcal) SAg exposure. This psoriatic disorder characteristically worsens with episodes of streptococcal pharyngitis and is ameliorated by anti-streptococcal prophylaxis.^[2]^ Our patient's daughter suffers from severe guttate psoriasis and has been offered anti-streptococcal therapy as well as intermittent IgG infusions in an effort to resolve her disfiguring condition. We suggest that the coincidence of her illness with that of her father results from a genetic inability to produce inhibitors, possibly IgG, to pyrogenic toxin SAgs. If so, therapy with intravenous IgG should be useful.

We conclude that “ SAgs cause super trouble” and that the presented case with its therapeutic solution exemplifies “insight-based medicine.”

## Author contributions

**Conceptualization:** Harry S. Jacob, Gregory M. Vercellotti, Donald Y.M. Leung, Patrick M. Schlievert.

**Data curation:** Harry S. Jacob, Gregory M. Vercellotti, Donald Y.M. Leung.

**Formal analysis:** Harry S. Jacob, Gregory M. Vercellotti, Donald Y.M. Leung.

**Funding acquisition:** Harry S. Jacob, Gregory M. Vercellotti, Donald Y.M. Leung.

**Investigation:** Harry S. Jacob, Gregory M. Vercellotti, Donald Y.M. Leung.

**Methodology:** Harry S. Jacob, Gregory M. Vercellotti, Donald Y.M. Leung, Patrick M. Schlievert.

**Project administration:** Harry S. Jacob, Gregory M. Vercellotti, Patrick M. Schlievert.

**Resources:** Harry S. Jacob, Gregory M. Vercellotti, Donald Y.M. Leung, Patrick M. Schlievert.

**Supervision:** Harry S. Jacob, Gregory M. Vercellotti, Patrick M. Schlievert.

**Validation:** Harry S. Jacob, Gregory M. Vercellotti, Patrick M. Schlievert.

**Visualization:** Harry S. Jacob, Gregory M. Vercellotti.

**Writing – original draft:** Harry S. Jacob, Gregory M. Vercellotti, Donald Y.M. Leung, Patrick M. Schlievert.

**Writing – review and editing:** Harry S. Jacob, Gregory M. Vercellotti, Donald Y.M. Leung, Patrick M. Schlievert.

Patrick M Schlievert orcid: 0000-0001-8314-9369.

## References

[1] Spaulding AR Salgado-Pabón W Kohler PL Staphylococcal and streptococcal superantigen exotoxins. Clin Microbiol Rev 2013;26:422–47. 23824366 10.1128/CMR.00104-12 PMC3719495

[2] Yarwood JM Leung DY Schlievert PM Evidence for the involvement of bacterial superantigens in psoriasis, atopic dermatitis, and Kawasaki syndrome. FEMS Microbiol Lett 2000;192:1–7. 11040420 10.1111/j.1574-6968.2000.tb09350.x

[3] Kotzin BL Leung DY Kappler J Superantigens and their potential role in human disease. Adv Immunol 1993;54:99–166. 8397479 10.1016/s0065-2776(08)60534-9

[4] Abe J Kotzin BL Jujo K Selective expansion of T cells expressing T-cell receptor variable regions V beta 2 and V beta 8 in Kawasaki disease. Proc Natl Acad Sci U S A 1992;89:4066–70. 1315049 10.1073/pnas.89.9.4066 PMC525633

[5] Leung DY Meissner HC Fulton DR Toxic shock syndrome toxin-secreting Staphylococcus aureus in Kawasaki syndrome. Lancet 1993;342:1385–8. 7901681 10.1016/0140-6736(93)92752-f

[6] Todd J Fishaut M Kapral F Toxic-shock syndrome associated with phage-group-I staphylococci. Lancet 1978;2:1116–8. 82681 10.1016/s0140-6736(78)92274-2

[7] Shands KN Schmid GP Dan BB Toxic-shock syndrome in menstruating women: association with tampon use and Staphylococcus aureus and clinical features in 52 cases. N Engl J Med 1980;303:1436–42. 7432402 10.1056/NEJM198012183032502

[8] Davis JP Chesney PJ Wand PJ The investigation and laboratory team. Toxic shock syndrome: epidemiological features, recurrence, risk factors, and prevention. N Engl J Med 1980;303:1429–35. 7432401 10.1056/NEJM198012183032501

[9] Schlievert PM Shands KN Dan BB Identification and characterization of an exotoxin from Staphylococcus aureus associated with toxic-shock syndrome. J Infect Dis 1981;143:509–16. 6972418 10.1093/infdis/143.4.509

[10] Jackow CM Cather JC Hearne V Association of erythrodermic cutaneous T-cell lymphoma, superantigen-positive Staphylococcus aureus, and oligoclonal T-cell receptor V beta gene expansion. Blood 1997;89:32–40. 8978274

[11] Leung DY Schlievert PM Meissner HC The immunopathogenesis and management of Kawasaki syndrome. Arthritis Rheum 1998;41:1538–47. 9751085 10.1002/1529-0131(199809)41:9<1538::AID-ART3>3.0.CO;2-M

[12] Leung DY Meissner HC Fulton DR Superantigens in Kawasaki syndrome. Clin Immunol Immunopathol 1995;77:119–26. 7586718 10.1006/clin.1995.1134

[13] Leung DY Meissner HC Shulman ST Prevalence of superantigen-secreting bacteria in patients with Kawasaki disease. J Pediatr 2002;140:742–6. 12072880 10.1067/mpd.2002.123664

[14] Leung DY Sullivan KE Brown-Whitehorn TF Association of toxic shock syndrome toxin-secreting and exfoliative toxin-secreting Staphylococcus aureus with Kawasaki syndrome complicated by coronary artery disease. Pediatr Res 1997;42:268–72. 9284264 10.1203/00006450-199709000-00004

